# Understanding Latino Individual and Family Perspectives in a National Diabetes Prevention Program

**DOI:** 10.1001/jamanetworkopen.2026.4780

**Published:** 2026-04-02

**Authors:** Fatima A. Tensun, Lilia Cervantes, Karen A. Uvina, Christine C. Welles, Nancy Wittmer, Jayna DeRoeck, Diana Pineda, Rocio I. Pereira

**Affiliations:** 1 Denver Health and Hospital Authority, Denver, Colorado; 2 Department of Medicine, University of Colorado Anschutz Medical Campus, Aurora

## Abstract

**Importance:**

Latino/a adults in the US are at higher risk for developing type 2 diabetes and are less likely to achieve goals in the National Diabetes Prevention Program (NDPP) than non-Latino/a White adults. Little is known about the barriers and facilitators to diabetes prevention behaviors among Latino/a adults.

**Objective:**

To describe the perspectives of Latino/a NDPP participants regarding barriers and facilitators to diabetes prevention behaviors.

**Design, Setting, and Participants:**

This qualitative study using semistructured phone interviews with Spanish-speaking Latino/a adults who participated in a community-based NDPP in an urban and suburban area of Colorado was conducted from June 2022 to August 2023.

**Main Outcome and Measures:**

The primary outcome was themes and subthemes reflecting individual and family support perspectives. Interviews were audio-recorded and transcribed. An inductive thematic analysis was conducted.

**Results:**

In total, 22 Spanish-speaking Latino/a participants, including 17 women (77%) and 5 men (22%) with a mean (SD) age of 44 (8) years, were interviewed. The interviews revealed 5 themes with respective subthemes. The first theme and subthemes described the perceived impact of culture and self-perception (frustration and presence of self-critical beliefs, perceived traditional gender roles, and internalized cultural biases of health behaviors) on diabetes prevention behaviors. The next 2 themes described family as reciprocal catalysts for diabetes prevention action (perceived family exercise engagement, dietary education influencing family eating habits, and collective emotional impact and accountability) and having a culturally and linguistically aligned program to support participants (perceived support and motivation from community health workers, culturally and linguistically concordant information as essential, and perceived peer support through shared experiences). The last 2 themes described reflection on personal motivation for behavior change (family diabetes lived experience awareness and motivation, desire to model lifestyle behaviors, and perceived mental health and spiritual appreciation) and desired program components for family inclusion (perceived role of knowledge for family participation, structured family-friendly wellness activities, and preventing diabetes among children).

**Conclusions and Relevance:**

In this qualitative study of Spanish-speaking Latino/a adults, participating in a diabetes prevention program, gender roles and internalized cultural biases were perceived as barriers to engaging in diabetes prevention behaviors, while family support and culturally aligned programming served as key facilitators. These findings may be used to improve diabetes prevention programs in Latino communities.

## Key Points

**Question:**

What are the perceived individual and family barriers and facilitators to lifestyle change for Latino/a adult participants in the National Diabetes Prevention Program?

**Findings:**

In this qualitative study of 22 Spanish-speaking Latino/a adults, participants described internal cultural biases and gender roles as barriers while highlighting family and program support as facilitators that influence diabetes prevention efforts.

**Meaning:**

These findings suggest that addressing cultural bias and gender roles and enhancing family and program support may improve future diabetes prevention programs, facilitating diabetes prevention behaviors among participating Latino/a adults.

## Teaser

This qualitative study examines perspectives of Latino/a individuals who participated in the National Diabetes Prevention Program regarding barriers and facilitators to diabetes prevention behaviors.

## Introduction

In the US, Latino/a adults are more likely than non-Latino/a White adults to have prediabetes,^1^ develop diabetes in their lifetime, experience diabetes complications,^2^ and die from diabetes-related causes.^3,4,5,6,7^ These disparities are largely due to structural and social determinants of health including differences in access to income and wealth, health care services, formal education, healthy foods, and healthy environments. Latino/a immigrants, who in 2024 made up one-third of the 68 million Latino/a individuals living in the US, are likely to face additional challenges to health such as low English language proficiency and lack of health insurance. Efforts to develop and disseminate effective diabetes prevention strategies for Latino/a immigrants have the potential to reduce health disparities and to inform similar strategies for other vulnerable groups.^3,4,5,6,7^

The National Diabetes Prevention Program (NDPP), established by the Centers for Disease Control and Prevention in 2010, is an evidence-based lifestyle intervention program that can delay or prevent diabetes among those at risk. Although the NDPP has been culturally adapted for Spanish-speaking adults through program delivery by community health workers (CHWs) who provide individualized support, tailored diet information, grocery shopping assistance, and diabetes education at the appropriate health literacy and education level,^8,9^ overall participation among Latino/a adults has been low.^10,11^ Moreover, Latino/a participants in the NDPP are less likely than non-Latino/a White participants to achieve the minimum 5% weight loss recommended for decreased future diabetes risk.^10,11^ A better understanding of facilitators and barriers to healthy behavior change among Latino/a participants in the NDPP is needed to support more effective diabetes prevention efforts for this population.

Previous studies of Latino/a individuals who already have a diagnosis of diabetes have found that family involvement, values, and peer support are essential components that reduce challenges to effective diabetes care. However, the perceived impact of family on diabetes prevention behaviors among Spanish-speaking Latino/a individuals participating in the NDPP remains largely unexplored.^12,13,14,15,16,17,18^ The core value of prioritization of family significantly influences health behaviors within this community.^12,13,19,20^ Although cultural values and norms are diverse and dynamic, both within and across ethnic and racial populations, prioritization of family continues to be particularly relevant for low-income Latina women who often bear the responsibilities of caregiving and food preparation.^21,22^

In our recent quantitative analysis of a longstanding community-based, culturally tailored NDPP intervention, we found high attendance was associated with higher rates of weight loss, yet only 30% of participants achieved the weight-loss goal.^23^ Furthermore, although attendance among men was lower than among women, men were more likely to achieve the recommended 5% weight loss goal.^23^ We hypothesized that family involvement, values, and peer support serve as facilitators to healthy behavior change among participants in the NDPP.

To our knowledge, no prior studies have explored the experiences and perspectives of the Latino community participating in the NDPP, particularly in relation to family involvement. We conducted a qualitative study to elucidate the perspectives of Latino/a men and women participants in a Spanish-language community-based NDPP.

## Methods

### Study Design

This qualitative study was approved by the Colorado Multiple Institution Review Board and follows the Consolidated Criteria for Reporting Qualitative Research (COREQ) reporting guideline.^24^ Semistructured one-to-one phone interviews were conducted between June 2022 and August 2023. Participants provided verbal consent and were compensated with a $25 gift card (see eAppendix 1 in Supplement 1 for more information on informed consent).

### Settings and Participants

Eligible participants self-identified as Latino/a or Hispanic, were aged 18 years or older, were English- or Spanish-speaking, and had completed the NDPP in the past 6 months. The NDPP was delivered by culturally and language-concordant CHWs employed by a Latino-serving community-based organization. Recruitment of study participants was completed using convenience sampling due to limited accessibility of the target population. CHWs identified eligible participants through their administrative database, and study personnel (F.T. and K.U.) contacted eligible participants via phone.

### Interview Guide

The interview guide was informed by a literature review of family health engagement and health behavior models and was designed to elicit facilitators and barriers to lifestyle changes in Latino/a adults participating in a culturally tailored diabetes prevention program.^16,17,25,26,27^ The interview guide used open-ended questions to explore perspectives related to individual NDPP experiences and family support. (eAppendix 2 in Supplement 1).

### Data Collection

Interviews were conducted by Latina bilingual research professionals with qualitative research expertise (F.T. and K.U.). Phone interviews were audio-recorded in Spanish, transcribed verbatim, and then professionally translated to English (by F.T. and K.U.) and deidentified. Accuracy of translations was verified by F.T., K.U., and R.P. (certified bilingual medical practitioners). The English translations were used for coding. Interviews were conducted until thematic data saturation (ie, when further observation and analysis did not reveal new concepts).^28,29,30^ Saturation was evaluated (by F.T. and K.U.) through ongoing, iterative analysis, with interviews reviewed sequentially to assess the emergence of new codes or themes. Data collection concluded when multiple consecutive interviews yielded no new thematic content.

### Data Analysis

ATLAS.ti version 23.2.1 (ATLAS.ti Scientific Software Development Gmbh) was used. Three authors (F.T., C.W., and R.P.) performed line-by-line coding, inductively identified and coded concepts, and grouped similar concepts into themes and subthemes through thematic analysis.^28,31,32,33^ Consensus on the themes and subthemes was reached through an iterative, thematic analysis by 4 coauthors: Latina physician researchers (R.P. and L.C.) and personnel with expertise in community-based programming and qualitative research (N.W. and K.U.). The team conducted peer debriefing to support analysis of coded data, engaged in iterative refinement, and resolved divergent perspectives during analysis until consensus was achieved. Investigator triangulation ensured that the themes reflected the full range and depth of the data. Member checking (returning data to participants for accuracy) was not performed due to limited participant availability.^34^

## Results

Thirty individuals were contacted via phone, and 22 individuals participated (17 women [77%] and 5 men [22%]; mean [SD] age, 44 [8] years) (Table 1). Reasons for nonparticipation included limited time (2 individuals), no interest (1 individual), and unresponsive to contact (5 individuals). All participants self-reported race and ethnicity as White and Hispanic or Latino/a. All participants reported being uninsured, and 16 (73%) were enrolled in a financial assistance program through the state. All interviews were conducted in Spanish. The mean (SD) interview duration was 27 (5) minutes. We identified 5 themes. One theme explored barriers of healthy behavior change, 2 themes focused on support influencing motivation and health facilitation, and 2 themes discussed participants’ outlook on health and desire for future program components of family inclusion. Themes and respective subthemes with supporting quotations translated into English are provided in Table 2. eAppendix 3 in Supplement 1 contains the original Spanish quotations.

**Table 1.** Characteristics of Study Participants

Characteristics	Participants, No. (%) (N = 22)
Demographic characteristics	
Age, mean (SD), y	44 (8)
Sex	
Female	17 (77.2)
Male	5 (22.8)
Preferred language for interview: Spanish	22 (100)
Marital status	
Married	19 (86.4)
Single	3 (13.6)
Race: White	22 (100)
Ethnicity: Hispanic or Latino/a	22 (100)
Socioeconomic characteristics	
Formal education	
Completed high school	13 (59.0)
<High school	9 (41.0)
Household size	
2-3 Members	6 (27.3)
4-5 Members	14 (63.6)
>6 Members	2 (9.1)
Household income, $	
<25 000	10 (45.5)
25 000-50 000	12 (54.5)
Health care and programmatic characteristics	
Primary care physician	
Yes	16 (72.7)
No	6 (27.3)
Insurance status or financial assistance	
Uninsured	22 (100)
Statewide financial assistance program	16 (72.7)
No financial assistance	6 (27.3)
Mode of program participation	
Virtual	16 (72.7)
Hybrid (in-person and virtual)	6 (27.3)

**Table 2.** Themes and Subthemes of Participants

Themes and subthemes	Quotations^a^
Theme 1: Perceived impact of culture and self-perception barriers on diabetes prevention	
Frustration and presence of self-critical beliefs	“[Yes, I did feel frustrated] when I had started losing weight but then gained it again...like in those moments when [I ate] fast food, like a burger or something like that. I failed. It’s hard for me to control my cravings and yes, I felt frustrated.” (Participant 2, Female)
	“I have to decrease [use of] some [sugary foods] because that helps lowers the [blood glucose] levels. We eliminated [some foods] completely. That’s where I was frustrated. Well one gets frustrated, because [it is difficult to change] the diet we already have.” (Participant 17, Male)
	“I would try to do all the program recommendations, but I could not lower my weight and blood sugars. That’s when I felt the most frustrated.” (Participant 9, Female)
	“I couldn’t overcome my temptations…like sodas and desserts. I always needed something sweet. There was that frustration of starting all over again. I got discouraged.” (Participant 11, Female)
Perceived traditional gender roles	“It’s not that I’m a chauvinist…but…I don’t see myself doing aerobic exercises. No, I am better doing things I like or something like that…riding a bicycle or something like that, but a hula hoop—this plastic ring they had there—that is not [something I like]. Well, no, I didn’t see myself doing that.” (Participant 3, Male)
	“Something that [my family] could help most with is to continue with diet changes. My wife cooks for everyone and we are trying to take out some [unhealthy foods]. There are times when one starts a [healthy habit] and after a few months we end up forgetting about it…or we get tired of the same routine. We go back to our old habits.” (Participant 22, Male)
	“I have personally had experiences, and with other mothers that both cook and work, that say they want to give up [making healthy changes]. I get out of work, and I try to find things to buy that are healthy and that my family likes. I mean, when it’s like that, I think it’s hard to make a change.” (Participant 10, Female)
	“My husband signed up for the program but with his job he couldn’t attend the classes. But I would make him healthy meals, so I had to keep up [with diet changes] for the both of us.” (Participant 1, Female)
Internalized cultural biases of health behaviors	“My daughter and I go to the gym. And it’s something that I had stopped doing in the past. I had done it before but then I stopped. In our culture, it is not something normal to go to the gym and do exercise.” (Participant 8, Female)
	“In our culture, it’s not really normal to go to the park. It can be tiring but it’s something so easy, but our culture does not usually do it. Not even exercise. Who knows how much time we have lost in our culture by not making those [healthy] changes?” (Participant 1, Female)
	“Making changes is more difficult for the whole family, especially here, [in the US] where the lifestyle is very fast paced, very accelerated. You have to keep working to have things [basic needs]. To live here. It’s hard.”(Participant 10, Female)
	“As Hispanics, Latinos, some of us do not want to go to the doctor unless we are sick, and we really do not exercise. It’s not in our culture.” (Participant 17, Male)
Theme 2: Family as reciprocal catalysts for diabetes prevention	
Perceived family exercise engagement	“For example, my daughter, my grandchildren, for example…they gifted me a mat to do yoga, with the names of my grandchildren and their little hands on it…to motivate me to do exercises because I don’t like exercising.” (Participant 5, Female)
	“I would do the exercises. My girl, the little one, would be beside me and the [community health workers] would say that we can all do it. She would be by my side and would watch. I liked the classes; it is truly something I would have never done…to exercise.” (Participant 7, Female)
	“What we [participant’s family] do and we now do is go outside and walk together. We go to the mountains, and we walk and ride bikes.” (Participant 3, Male)
	“Walking is something we now do as a family. [We go] to the park, and we are also doing activities that involve food. All of us are doing all those things together now.” (Participant 22, Male)
	“Yes we could come to go out walk all together. At the end of the week, go to the park or something. But right now, it is a bit complicated to do it together.” (Participant 4, Female)
Dietary education influencing family eating habits	“Initially my family didn’t like the healthy food I would get. But I have always added the new food [healthy food] in their meals, but I would wait. Then slowly I would add more in their meal.” (Participant 2, Female)
	“Well, my kids knew that I was participating in the classes, and they also would remind me—remember [you are not supposed to eat that]. When we would go to the store, and we would grab cookies for example, they would say ‘no because it has too much sugar.’ They [participant’s children] would help us choose our food items.” (Participant 1, Female)
	“Well, we [participant’s family] had this custom of making tamales. So, instead of eating just tamales, we would make the tamales much thinner... very tiny…pure dough, and we would work to add more vegetable or reduce portions. For example, having the tamal and adding a lettuce and tomato salad so it wasn’t just the tamales.” (Participant 12, Female)
	“My kids and I shop together and read labels. I would say too, on occasion my kids have helped me make new [healthy] recipes.” (Participant 11, Female)
	“I tried to involve him [participant’s spouse] too, but he does not want to eat healthy. He says it’s too difficult to keep up with.” (Participant 6, Female)
Collective emotional impact and accountability	“I feel grateful that my wife supported me too. She doesn’t have any illnesses, but she supported me by being with me when signing up and taking classes together.” (Participant 18, Male)
	“My children ask me; did you take dad to [NDPP] class too? We are going to make changes here! We have wanted to this for a while. It’s not easy but we are trying together. I feel happy.” (Participant 14, Female)
	“[My family] are my support because I don’t like going out by myself. It feels too lonely; but when they are with me, I can [go out and] do my exercises.” (Participant 12, Female)
	“Currently, there is a complication with our work [participant and participant’s spouse] schedule to keep up healthy habits. But yes, it feels good to get encouragement to continue.” (Participant 9, Female)
Theme 3: Culturally and linguistically aligned program to support participants	
Perceived support and motivation from community health workers	“Yes, because with whatever question we had, we [participants] would ask ourselves and well, they [community health workers] would be there...teaching us and supporting us.” (Participant 6, Female)
	“I learned to talk through my frustration in the program. All anyone needs is someone to listen to them. In the same way, it helps other people in the program.” (Participant 15, Female)
	“I feel more reassured by them, to ask questions more directly to them. It is easier to understand [what someone is teaching] when they are looking at you and listening to you.” (Participant 2, Female)
	“When we (participants) finished the program, there was follow up every month. It was pretty consistent. From there, I was pretty motivated, but when there were no classes with them [community health workers], the meetings were more far out. I started to decline again. I got discouraged and did not exercise anymore.” (Participant 13, Female)
Culturally and linguistically concordant information as an essential	“Yes, [community health workers] worked very hard with us to teach us how to buy food in the market and read labels like foods that have too much sugar.” (Participant 19, Male)
	“They [community health workers] taught us how to prepare food, but in a healthier way…so yes, it helped me, because it gave me options to think and understand—I can eat this, but I can make it healthy.” (Participant 11, Female)
	“They (community health workers) gave us good tips for when we go to the store, like reading labels and counting calories.” (Participant 12, Female)
	“They gave information…the most important was how diabetes was caused, who gets it? The differences between diabetes and prediabetes. Well, they explained to us in a way that was very easy to understand.” (Participant 9, Female)
	“There should be more information said to the rest of the family for more clarity. Label reading was hard for me to explain.” (Participant 7, Female)
Perceived peer support through shared experiences	“It was also very helpful to have the other participants there. They also shared their experiences. We truly feel like a community that supports one another.” (Participant 18, Male)
	“There were people like me in the program...trying to do the same things. Well, we all had the same conditions, and we could do it...supporting one another to continue.” (Participant 8, Female)
	“I listened to other people that have similar problems as me and I realized that it’s not just me [I am not alone]. I can do it.” (Participant 20, Female)
	“We started doing the nutrition but I was not losing weight and some other peers were losing weight. And some of us were the same [weight]. So that was difficult.” (Participant 16, Female)
Theme 4: Reflection on personal motivation for behavior change	
Family diabetes lived experience awareness and motivation	“My mom had diabetes. I have a lot of family with diabetes and majority of my sisters are diabetic because they don’t take care of themselves. I have to be healthy.” (Participant 14, Female)
	“My husband’s mother has diabetes, right? So, we should try to improve [our health] because we are prone to diabetes. If we don’t take care of ourselves, later we will also develop diabetes. So, we should try together.” (Participant 20, Female)
	“I participated because my family all have diabetes. The majority of my siblings carry a family history of diabetes…in other words, I do care about my health. The most important thing is health.” (Participant 17, Male)
	“I saw my mom suffer from diabetes and eventually it took her life. So yes, that motivates me.” (Participant 6, Female)
Desire to model lifestyle behaviors	“I am the one who cooks. They [participant’s family] eat what I try to eat...like vegetables.” (Participant 4, Female).
	“I want to be a good example for them even if I’m the grandmother, I can be healthy. I can make changes. It does not matter what age I am to be active...a good example for them.” (Participant 5, Female).
	“I want to continue doing exercise and having a healthy lifestyle to support my kids. An example is that, if I want to change that in my kids, I need to start first, and in way we support each other, helping one another...” (Participant 8, Female)
	“I didn’t want to do it for me but for my family. Eventually we helped each other resist eating junk food.” (Participant 12, Female).
	“We are working together [participant and participant’s spouse]. But with him [participant’s spouse], he gave up. He did not want to eat healthy anymore. He tried to again but then he ate more tortillas. Me? No. I make my own food. He started over with that [healthy eating].” (Participant 1, Female)
Perceived mental health and spiritual appreciation	“[From the NDPP] I learn to tell myself and at the same time make them [participant’s family] aware that if they continue with a poor diet, they will have health risks. But we all feel mentally good doing things together.” (Participant 21, Male)
	“The truth is that sometimes, we as humans do not want to understand [health] and we don’t realize—we can prevent diseases if we really put our minds into it. Sometimes we might fall mentally but one needs to ask God to help us get through it. Thankfully I overcame those [diabetes-related] challenges.” (Participant 15, Female)
	“Something positive that continues to motivate me is yoga and having family there for me. The best part was that they [participant’s family] were always there to keep me fit and it helped me a lot mentally.” (Participant 10, Female)
	“When one falls behind on that [continuing healthy habits]. How does one feel? Well depressed or something. And you feel like you can’t do it. You want to go back to your old life with bad food, diet, and things like that.” (Participant 16, Female)
Theme 5: Desired program components for family inclusion	
Perceived role of knowledge for family participation	“I would have wanted to get more involved with understanding the problems [diabetes]….I was taking classes, and I would explain them to my husband too. But he would have understood more listening in the class.” (Participant 6, Female)
	“Well, more than anything, I want them [participant’s family] to be informed. Because they told me that I was already informed. It is not the same for me to come and tell them the information.” (Participant 22, Male).
	“We need to all understand why we should eat healthy food. As well as learning that some foods may harm us too.” (Participant 19, Male)
	“The only way your family can help you is by becoming aware, that is, informing themselves so they become aware of diabetes too.” (Participant 5, Female)
	“One of those sessions, they should have invited the family. It would have been great if they were invited to get that information.” (Participant 12, Female)
Structured family-friendly wellness activities	“We want to do a lot of exercise. My girls try to stay active going to the gym. They go more than me, because the problem for me is having time, and where I live. I go to Zumba classes. But it would be nice to do it together.” (Participant 14, Female)
	“We [participant’s family] want to do more in the summer when it gets warmer…to go to the park more, bike more. I want to continue with exercising to continue with my goals and stay active.” (Participant 4, Female)
	“I would like my husband to be more involved and accompany me to exercise [but] I think, I can’t force a person to do what I want, right?” (Participant 8, Female)
	“ Yes, we [participant’s family] could come to go out walk all together at the end of the week go to park or something. But now is a bit complicated to do it together.” (Participant 6, Female)
Preventing diabetes among children	“I hope one day I can sign up my children for the program. Including them is key, so we can say that we are all in it together, right?” (Participant 11, Female)
	“I would like my kids to be motivated and learn a bit about their snacks.” (Participant 10, Female)
	“The [diabetes program] had the program for young children, but they don’t have it anymore. But I would like it to be more of that like nutrition…classes, because I learned a lot there.” (Participant 7, Female)
	“Our children are always learning, and I always try to teach them things. I hope in the future they can also teach this program to kids, and teach them things like eating healthy, doing exercise, and walking. All of that is important.” (Participant 1, Female)
	“Well when I was a little girl…You eat when you’re filled up and having that habit is hard to take away. You get full until you are satisfied. Teaching that to kids may be hard.” (Participant 13, Female)

Abbreviation: NDPP, National Diabetes Prevention Program.

^a^ Quotations from interviews were conducted in Spanish and translated in English by Spanish-speaking study personnel. eAppendix 3 in Supplement 1 contains the original Spanish quotations.

### Theme 1: Perceived Impact of Culture and Self-Perception Barriers on Diabetes Prevention

#### Frustration and Presence of Self-Critical Beliefs

Participants reflected on their feelings of frustration when it came to diabetes prevention. They reported difficulty sustaining diabetes prevention actions as well as frustration when their actions did not translate to concrete clinical outcomes including weight loss and lower blood glucose. One participant stated, “I would try to do all the program recommendations, but I could not lower my weight and blood sugars. That’s when I felt the most frustrated” (Participant 9, Female). Additionally, participants had self-critical views related to their diabetes prevention actions. One participant expressed, “I couldn’t overcome my temptations…I always needed something sweet. There was that frustration of starting all over again. I got discouraged” (Participant 11, Female).

#### Perceived Traditional Gender Roles

Participants described traditional gender roles that limited them from performing diabetes prevention activities. Women participants reported that they were the primary cook and stayed at home to parent; they stated that their male partner was the primary breadwinner for the household. Other women participants reported the difficulties of being a working mother and having limited time to shop and cook. They also experienced challenges cooking healthy meals that all family members, particularly the children, would eat. Some women reported having to cook separate meals for themselves if their husband and/or the children did not accept the meal modifications they tried to make. Women whose husbands were not able to attend the classes experienced an added responsibility to make changes on behalf of their spouse in addition to making changes for themselves. One female participant stated, “My husband signed up for the program but with his job he couldn’t attend the classes. …I had to keep up [with diet changes] for the both of us” (Participant 1, Female). These experiences were not shared by men participants.

Most men reported that their wives prepared the meals, and they experienced diet changes as being not entirely in their control. One male participant stated, “Something that [my family] could help most with is to continue with diet changes. My wife cooks for everyone and we are trying to take out some [unhealthy foods]. There are times when one starts a [healthy habit] and after a few months we end up forgetting about it…or we get tired of the same routine. We go back to our old habits” (Participant 22, Male). Male participants also shared reluctance to engage in certain diabetes prevention activities viewing them as too feminine. One male participant stated, “It’s not that I’m a chauvinist…but…I don’t see myself doing aerobic exercises. No, I am better doing things I like…riding a bicycle or something like that…” (Participant 3, Male)

#### Internalized Cultural Biases of Health Behaviors

Participants reflected on cultural biases that shape their health behaviors in diabetes prevention. They described how their Latino culture does not value healthy habits like exercising, attributing their internal cultural health biases as a barrier for diabetes prevention. One participant stated, “As Hispanics, Latinos, some of us do not want to go to the doctor unless we are sick, and we really do not exercise. It’s not in our culture” (Participant 17, Male).

### Theme 2: Family as Reciprocal Catalysts for Diabetes Prevention Action

#### Perceived Family Exercise Engagement

Participants expressed that once they learned about healthy habits through the program, they and their families began to modify their routines. These changes included incorporating exercise into their daily activities as a family, and they shared their motivation to engage in family exercise activities. One participant stated, “Walking is something we now do as a family. [We go] to the park, and we are also doing activities that involve food. All of us are all doing all those things together now” (Participant 22, Male).

#### Dietary Education Influences Family Eating Habits

Participants expressed that after learning about nutrition and making dietary changes, their family members began doing the same. One participant stated, “Everyone changed their diet, even our grandchildren did” (Participant 5, Female). Other participants reported behavioral changes including engaging in food-related activities such as shopping with their family members. “My kids and I shop together and read labels. I would say too, on occasion my kids have helped me make new [healthy] recipes” (Participant 11, Female).

#### Collective Emotional Impact and Accountability

There was increased shared accountability and engagement to do activities together while participating in the NDPP. A participant stated, “My children ask me, did you take dad to [NDPP] class too? We are going to make changes here! We have wanted to this for a while. It’s not easy but we are trying together…I feel happy doing [activities] with [the family]” (Participant 14, Female). While participants reported that making healthy behavior changes as a family made the changes easier, some also reported that changes were not maintained after loss of family engagement. For instance, participants reported becoming more active when the entire family was able to exercise together, then stopping exercise when schedules no longer aligned. Thus, shared accountability did not lead to self-accountability, and loss of family support was experienced as a barrier to maintaining healthy behaviors that were started as a family.

### Theme 3: Culturally and Linguistically Aligned Program to Support Participants

#### Perceived Support and Motivation From Community Health Workers

Participants described support from CHWs, who fostered a positive environment that motivated participants to make behavior changes. Participants described how CHWs motivated them to believe that they can prevent diabetes. A participant stated, “I feel more reassured by them, to ask questions more directly to them. It is easier to understand [what someone is teaching] when they are looking and listening to you” (Participant 2, Female).

#### Culturally and Linguistically Concordant Information as an Essential

Participants attributed their increased understanding of diabetes prevention actions to CHWs delivering information in an accessible way, matching their health literacy level, and allowing integration into daily routines. For example, they could watch home exercise videos recorded by CHWs or apply lessons during routine tasks like grocery shopping. A participant stated, “They [CHWs] gave us good tips for when we go to the store, like reading labels and counting calories” (Participant 12, Female). Another participant said, “They gave information…the most important was how diabetes was caused, who gets it? …the differences between diabetes and prediabetes. Well, they explained it to us in a very easy way to understand” (Participant 9, Female).

#### Perceived Peer Support Through Shared Experiences

Participants described how peer support through shared experiences with similar challenges was helpful in diabetes prevention. One participant stated, “I listened to other people that have similar problems as me and I realized that it’s not just me [I am not alone]. I can do it” (Participant 20, Female). Another participant stated, “It was also very helpful to have the other participants there. They also shared their experiences too. We truly feel like a community that supports one other” (Participant 18, Male). Other participants reported comparing themselves with peer participants and viewed this as a barrier. One participant stated, “We started doing the nutrition but I was not losing weight and some other peers were losing weight. And some of us were the same [weight]. So that was difficult” (Participant 16, Female).

### Theme 4: Reflection on Personal Motivation for Behavior Change

#### Family Diabetes Lived Experience Awareness and Motivation

Participants described the importance of awareness of long-term consequences of unhealthy habits. Participants shared that their families were impacted by diabetes and how this motivated them to continue to engage in diabetes prevention activities. One participant said, “I saw my mom suffer from diabetes and eventually it took her life. So yes, that motivates me” (Participant 6, Female).

#### Desire to Model Lifestyle Behaviors

Participants expressed that their role as a parent motivated them to engage in diabetes prevention activities so that they could stay healthy for their family. Participants also described their desire to be a good role model for their family. One participant reflected, “I didn’t want to do it for me but for my family. Eventually we helped each other resist eating unhealthy foods” (Participant 12, Female).

#### Perceived Mental Health and Spiritual Appreciation

Participants reported changes in physical and mental health when family was involved in diabetes prevention. One participant shared, “Something positive that continues to motivate me is yoga and having family there for me. The best part was that they [participant’s family] were always there to keep me fit and it helped me a lot mentally” (Participant 10, Female). Additionally, participants noted the importance of spirituality. One participant said, “Sometimes we might fall down mentally but one needs to ask God to help us get through it. Thankfully I overcame those [diabetes-related] challenges” (Participant 15, Female).

### Theme 5: Desired Program Components for Family Inclusion

#### Perceived Role of Knowledge for Family Participation 

Participants expressed desire for their family members to also receive information by being formally included in the diabetes prevention program. One participant stated, “The only way your family can help you is by becoming aware, that is, informing themselves so they become aware of diabetes too” (Participant 5, Female).

#### Structured Family-Friendly Wellness Activities

Participants expressed the desire for their family members to join in healthy eating and exercising activities together. One participant expressed the difficulty of having time conflicts of doing activities together: “We want to do a lot of exercise. My girls try to stay active going to the gym. They go more than me, because the problem for me is having time, and where I live. I go to Zumba classes. But it would be nice to do it together” (Participant 14, Female).

#### Preventing Diabetes Among Children

Participants expressed the desire to teach their children about healthy habits and wished to have a program component that includes their children, allowing them to participate as well. One participant stated, “Our children are always learning…I hope in the future they can also teach this program to kids, and teach them things like eating healthy, doing exercise, and walking. All of that is important” (Participant 1, Female).

## Discussion

This qualitative study explored the perspectives of Spanish-speaking Latino/a adults participating in an NDPP. Participants found that cultural factors and self-perceptions influence diabetes prevention efforts, creating barriers to family involvement and influencing healthy behavior adoption. Our study also revealed that family support can serve to mitigate these barriers, serving as a catalyst for healthy behaviors among participants as well as their family members. Additionally, support from NDPP staff and peers is crucial for maintaining participant motivation. These insights emphasize the importance of culturally tailored diabetes prevention programs to support successful engagement in the NDPP.

To our knowledge, this is the first study to investigate individual perceptions and family support experiences of Spanish-speaking Latino/a adults participating in the NDPP intervention. Participants reflected on their motivations and identified desired program components for family inclusion, which could inform efforts to improve diabetes prevention. Personal motivation included sharing their family history of type 2 diabetes to raise awareness about their risk, gaining knowledge to prevent diabetes, and teaching their children healthy habits. These findings are consistent with prior studies that have found values such as prioritization of family over the individual playing an important role in supporting healthy behavior change among Latino communities.^2,16,19,35,36^ Participants also emphasized family support as key in promoting healthy behaviors for diabetes prevention, aligning with studies that highlight the role of supporting Latino/a adults with type 2 diabetes through accountability and companionship.^15,20,36,37^ These findings suggest future interventions could integrate family dynamics into diabetes prevention to foster support aligned with participants’ values.

Furthermore, participants indicated that family members serve as reciprocal catalysts for diabetes prevention. These findings are consistent with studies that highlight how Latina women’s management of family’s health leads to healthy behaviors in the household.^15,19,38,39^ Participants shared that their food environments in the household changed as they learned about nutrition and healthy eating habits. Our study shows that collective emotional impact and accountability are also present in families, which is consistent with studies investigating diabetes self-management in the Latino population.^13,17,40,41,42,43^ The support from the NDPP staff and peers was shared among participants, displaying the power of person-centering—a construct of support to establish a safe space to create interpersonal connection and trust in health care.^44,45,46,47^ Participants reported feeling comfortable and empowered to share their experiences with CHWs and peers. Through NDPP classes with CHWs and peer support, we found that participants reported having a better understanding of diabetes prevention. These findings emphasize the importance of accessible and culturally aligned program information for understanding diabetes prevention, which is consistent with prior literature.^9,48,49,50,51^ Future interventions could prioritize culturally tailoring the program to improve engagement and effectiveness of diabetes prevention within Latino communities.

Our findings also highlight that, while support is crucial, cultural factors and self-perception can hinder family involvement. A novel finding was the perceived gender roles and cultural norms as potential barriers to diabetes prevention. Male study participants reported the activities were too feminine, while women reported that family caregiving responsibilities limited their time for diabetes prevention activities. Men also expressed that as the primary breadwinners, they felt they had the authority to accept or refuse the proposed changes. Women attempted integrating healthy changes in their routines, such as healthy cooking or shopping for their families but faced resistance from their spouse and children. Our findings are consistent with studies which found that Latino men self-report more traits traditionally associated with males (ie, not needing health care services and being primary breadwinners), which may lead to reluctance to engage in healthy behaviors.^13,19,20,52^ Likewise, with Latina women, studies highlight that their gender role in health behaviors is displayed through values traditionally associated with women, such as respect and deference to their husbands, being family-oriented, and being a primary caretaker.^12,14,17,53,54,55,56^ These findings provide insight on barriers to participation among men as observed in our recent program evaluation.^23^ They also suggest diabetes prevention programs could consider gender and cultural dynamics, encourage shared decision-making, and offer personalized support that integrates activities appealing to masculine and feminine preferences.

Finally, participant statements suggested internalized cultural biases led to deprioritizing health-seeking actions, such as regular doctor visits and maintaining a healthy lifestyle for diabetes prevention. Participants expressed that health-seeking behaviors—such as visiting the doctor, exercising regularly, and maintaining a healthy diet—are often not prioritized within Latino culture due to constraints like limited time and responsibilities. These findings are consistent with studies showing that shared cultural beliefs impact an individual’s health.^25,56,57,58,59^ Future diabetes prevention programs could implement strategies that personalize their efforts, such as fostering an open discussion of health-seeking behaviors and cultural beliefs, to better engage Latino communities in diabetes prevention.

### Limitations

The study has limitations. Participants may have given responses they thought program staff would want to hear, indicating possible social desirability bias. Recall bias may have been a limitation because participants completed the program more than 6 months before the interview. However, we focused on recruiting recent participants to minimize this effect. Use of convenience sampling and including only participants who completed the program may have introduced selection bias and limited generalizability. We do not have information regarding country of origin of the participants or how long they lived in the US, variables which may have impacted acculturation and influenced their experiences. Additionally, this sample included Spanish-speaking adults in a single community-based diabetes prevention program, all of whom were uninsured, and the majority of the whom were female, so the findings may not be generalizable to other populations.^60^

## Conclusions

This qualitative study of Spanish-speaking Latino/a individuals participating in a diabetes prevention program described perceived gender roles and internalized cultural biases as barriers to engaging in diabetes prevention behaviors. Participants valued culturally tailored program components and family support as key facilitators for health behavior change. Future diabetes prevention programs could emphasize family involvement and address cultural biases to better support Latino populations.

## Notes

Supplement 1. eAppendix 1. Post Card Consent, English and Spanish eAppendix 2. Interview Guide (English and Spanish) eAppendix 3. Spanish Quotes With English Translations

Supplement 2. Data Sharing Statement
