# Supplementary material for: Understanding Latino Individual and Family Perspectives in a National Diabetes Prevention Program
Source: JAMA Netw Open. 2026 Apr 2;9(4):e264780. doi: 10.1001/jamanetworkopen.2026.4780 (PMC13047464; doi:10.1001/jamanetworkopen.2026.4780)
Supplement: Supplement 1. — eAppendix 1. Post Card Consent, English and Spanish eAppendix 2. Interview Guide (English and Spanish) eAppendix 3. Spanish Quotes With English Translations [file jamanetwopen-e264780-s001.pdf]

Supplemental Online Content

Tensun FA, Cervantes L, Uvina KA. Understanding Latino individual and family perspectives in a national diabetes prevention program. *JAMA Netw. Open*. 2026;9(4):e264780. doi:10.1001/jamanetworkopen.2026.4780

eAppendix 1. Post Card Consent, English and Spanish
eAppendix 2. Interview Guide (English and Spanish)
eAppendix 3. Spanish Quotes with English Translations

This supplemental material has been provided by the authors to give readers additional information about their work.

© 2026 Tensun FA et al. *JAMA Network Open*.

eAppendix 1. Post Card Consent, English and Spanish

Study Title: Family Support in Vuela for Health-Diabetes Prevention Program (DPP) Participants
Principal Investigator: Mentor Principal Investigator: Rocio Pereira, MD , Student Principal Investigator: Fatima Tensun

COMIRB No:22-0494

Version Date: May 5th, 2022

You are being asked to be in a research study because you previously participated in the Diabetes Prevention Program (DPP) with Vuela for Health and are the ages between 18 and 88. This form provides you with information about the study.

If you join the study, you will be asked to participate in an interview over the phone about your experiences with the Diabetes Prevention Program (DPP) to better understand familial involvement and behavior. The interviews will be audio recorded by the interviewer to capture all your responses accurately. The interview will last anywhere between 30 minutes to 60 minutes. This study is a part of the researcher's Colorado School of Public Health project.

The study is designed to learn more about family support and experiences with the Diabetes Prevention Program (DPP) at Vuela for Health.

Possible discomforts or risks may include emotional experience as you reflect on your experience of diabetes prevention and familial involvement. We recognize that the information you share about your experience with the Diabetes Prevention Program (DPP) is sensitive and we will ensure to maintain your privacy in the interview. There may be risks the researchers have not thought of.

The study is not designed to benefit you directly.

Every effort will be made to protect your privacy and confidentiality by removing any personal identification from the data and assigning an identification number that will be uniquely associated with your recorded interviews.

You will be paid $25.00 for participating in the study. Payments for participating in this research study will be deposited on a "ClinCard". ClinCard cards are managed by a company called Greenphire. ClinCard cards work like a gift card and can be used at any store that accepts Mastercard. At a minimum, your name, date, and address will be provided to Greenphire for research study payments to be credited to the ClinCard. Please let the research staff know if you have questions about using the ClinCard. It is important to know that payments for participating in a research study are taxable income. If you receive $600 or more from the Denver Health & Hospital Authority in a tax year, an IRS Form 1099 will be sent to you for tax purposes. It will cost you nothing to be in the study.

© 2026 Tensun FA et al. *JAMA Network Open*.

You have a choice about being in this study. You do not have to be in this study if you do not want to be.

The data we collect will be used for this study but may also be important for future research. Your data may be used for future research or distributed to other researchers for future study without additional consent if information that identifies you is removed from the data.

If you have questions, you can call Fatima Tensun at 720-375-3274. You can call to ask questions at any time.

You may have questions about your rights as someone in this study. If you have questions, you can call COMIRB (the responsible Institutional Review Board) at (303) 724-1055.

By completing this interview, you are agreeing to participate in this research study.

© 2026 Tensun FA et al. *JAMA Network Open*.

Titulo del estudio: Apoyo Familiar para los participantes del Programa de Prevención de la Diabetes (DPP) de Vuela por Salud
Investigador Principal: Mentor Investigador Principal: Rocio Pereira, MD , Investigador Principal Estudiantil: Fatima Tensun
Núm. De COMIRB: 22-0494
Fecha de versión: 5 Mayo de 2022

Se le esta pidiendo que participe en un estudio de investigación porque anteriormente participó en el Programa de Prevención de Diabetes (DPP) con Vuela por Salud y tiene entre 18 y 88 años. Este formulario le proporciona información sobre el estudio.

Si se une al estudio, se le pedirá que participe en una entrevista por teléfono sobre sus experiencias con el Programa de Prevención de la Diabetes (DPP) para entender mejor la participación familiar y el comportamiento. Las entrevistas serán grabadas en audio por el entrevistador para capturar todas sus respuestas con precisión. La entrevista durará entre 30 y 60 minutos. Este estudio es parte del proyecto de la Escuela de Salud Pública de Colorado del investigador.

El estudio está diseñado para aprender más sobre el apoyo familiar y las experiencias con el Programa de Prevención de la Diabetes (DPP) en Vuela for Health.

Las posibles molestias o riesgos pueden incluir experiencia emocional al reflexionar sobre su experiencia de prevención de la diabetes y participación familiar. Reconocemos que la información que comparte sobre su experiencia con el Programa de Prevención de Diabetes (DPP) es privado y nos aseguraremos de mantener su privacidad en la entrevista. Puede haber riesgos que los investigadores no hayan pensado.

Este estudio no está diseñado para beneficiarlo directamente.

Se hará todo lo posible para proteger su privacidad y confidencialidad, al remover cualquier identificación personal a los datos y asignar un numero de identificación que será lo único que se relacionará con sus respuestas en las entrevistas grabadas.

Se le pagará $ 25.00 por participar en el estudio. Los pagos por participar en este estudio de investigación se depositaran una tarjeta "ClinCard". Las tarjetas ClinCard son administradas por una compañía llamada Greenphire. Las tarjetas ClinCard funcionan como una tarjeta de regalo y puede usarse en cualquier establecimiento que acepte Mastercard. Como un mínimo, su nombre, fecha de nacimiento y su dirección se le dará a Greenphire para que los pagos del estudio de investigación se acrediten en la tarjeta ClinCard. Informe al personal de investigación si tiene dudas sobre el uso de la tarjeta ClinCard. Es importante saber que los pagos por participar en un estudio de investigación son ingresos imponibles. Si recibe $ 600 o más de Denver Health & Hospital Authority en un año fiscal, se le enviará un Formulario 1099 del IRS para fines fiscales. No le costará nada estar en el estudio.

© 2026 Tensun FA et al. *JAMA Network Open*.

Tiene la opción de participar en este estudio. Usted no tiene que participar en este estudio de investigación si no desea participar.

Los datos que recopilamos se utilizarán para este estudio, pero también pueden ser importantes para futuras investigaciones. Sus datos pueden usarse para futuras investigaciones o distribuirse a otros investigadores para futuros estudios sin consentimiento adicional, si la información que lo identifica se elimina de los datos.

Si tiene preguntas, puede llamar a Fatima Tensun al 720-375-3274. Puede llamar para hacer preguntas en cualquier momento.

Es posible que tenga preguntas sobre sus derechos como persona en este estudio. Si tiene preguntas, puede llamar a COMIRB (la Junta de Revisión Institucional responsable) al (303) 724-1055.

Al completar esta entrevista, usted acepta participar en este estudio de investigación.

© 2026 Tensun FA et al. *JAMA Network Open*.

eAppendix 2. Interview Guide (English and Spanish)

Protocol #:22-0494
Version Date: 05.03.2022

Interview Guide (English)

Introduction:

I want to thank you for taking the time from your busy schedule to interview with me. This interview will be recorded. I will make every effort to protect your privacy and confidentiality by removing any personal information about you the project. Are there any questions before we begin?

1. What has been your experience with Diabetes Prevention Program?
   - **Probes**: Tell me more? What do you mean?

2. What were moments that you felt positive and proud?
3. What were moments that you felt frustrated?
4. Throughout your experience with the Diabetes Prevention Program, was anyone supporting you? Do they live with you?
   - **Probes**: Could you please tell me more about how they were involved? What feelings do you have when your family or friend is involved?
   - If participant did not have any family members involved: Why that is?

5. How has your family continue to be involved in your diabetes prevention today?
   - If participant did not have any family members involved: Is your family involved in your diabetes prevention journey today? If so, please explain how or why not.

6. What is something that you were able to change at home with family that you have not done after participating in the diabetes prevention program?
   - If participant did not have any family members involved: What is something that you were able to change at home that you have not done before after being in the diabetes prevention program.

7. What is something you wish your family was able to help for your diabetes prevention?
8. In the Diabetes Prevention Program, was there a component in the program that you wish you would have wanted family to be involved in?
9. Do you believe that family involvement is important to diabetes prevention?
10. What is your plan to continue following a healthy lifestyle for preventing diabetes?

Núm. De COMIRB: 22-0494

Fecha de versión: 3 Mayo de 2022

Guía de entrevistas (Español)

Introducción:

Quiero agradecerle por tomarse el tiempo de su apretada agenda para entrevistarse conmigo. Esta entrevista será grabada. Haré todo lo posible para proteger su privacidad y confidencialidad eliminando cualquier información personal sobre usted en el proyecto. ¿Hay alguna pregunta antes de comenzar?

1. Cómo ha sido su experiencia con el Programa de Prevención de la Diabetes?
   - Cuentame más? ¿Qué quiere decir?
2. Cuáles fueron los momentos en los que te sentiste positivo y orgulloso?
3. Cuáles fueron los momentos en los que te sentiste frustrado?
4. A lo largo de su experiencia con el Programa de Prevención de la Diabetes, Alguien le apoyó? Viven con usted?
   - Podría decirme más sobre cómo estuvieron involucrados? ¿Qué sentimientos tuvo cuando su familia or amigo está involucrada?
   - Si el participante no tuvo ningún miembro de la familia involucrado: Porqué?
5. Cómo ha seguido participando su familia en la prevención de su diabetes hoy en día?
   • Si el participante no tuvo ningún miembro de la familia involucrado: ¿Está su familia involucrada en su viaje de prevención de la diabetes hoy? Si es así, por favor explique cómo o por qué no.
6. Qué es algo que pudiste cambiar en casa con la familia que no has hecho después de participar en el programa de prevención de la diabetes?
   • Si el participante no tenía ningún miembro de la familia involucrado: ¿Qué es algo que pudo cambiar en casa que no ha hecho antes después de estar en el programa de prevención de la diabetes?
7. Qué es algo que usted desea que su familia pueda ayudar para su prevención de la diabetes?
8. En el Programa de Prevención de Diabetes, había algún componente en el programa en el que quisieras que la familia estuviera involucrada?
9. Cree que la participación familiar es importante para la prevención de la diabetes?
10. Cuál es su perspectiva futura en su prevención de la diabetes?

© 2026 Tensun FA et al. *JAMA Network Open*.

eAppendix 3. Spanish Quotes with English Translations

Theme 1: Impact of Culture and Self-Perception Barriers on Diabetes Prevention	
Subtheme: Frustration and presence of Self-Critical Beliefs	
**"Spanish Quote from Interview"**	**"English Translation"**
"Cuando ya estaba perdiendo peso y lo volvía a ganar… Sí, me sentí frustrada. Como en esos momentos en que vas por comida rápida, como una hamburguesa o algo así. Fallé. Es difícil para mí. Como controlar mis antojos y si, si me sentía frustrada. "	"[Yes, I did feel frustrated] when I had started losing weight but then gained it again... Like in those moments when [I ate] fast food, like a burger or something like that. I failed. It's hard for me to control my cravings and yes, I felt frustrated." (Participant 2, Female)
"Bueno, tengo que dejar fuera algunos azúcares porque eso ayuda a bajar los niveles. Si no, lo eliminamos por completo. Ahí es donde estaba frustrado. Bueno uno se frustra, porque la dieta que ya tenemos."	"I have to decrease [use of] some [sugary foods] because that helps lowers the [blood glucose] levels. We eliminated [some foods] completely. That's where I was frustrated. Well one gets frustrated, because [it is difficult to change] the diet we already have." (Participant 17, Male)
"Trataría de hacer todas las recomendaciones del programa, pero no podía bajar mi peso y los azúcares de la sangre. Fue entonces cuando me sentí más frustrada."	" I would try to do all the program recommendations, but I could not lower my weight and blood sugars. That's when I felt the most frustrated." (Participant 9, Female)
"No pude superar mis tentaciones. Como refrescos y dulces. Verdad. Siempre necesitaba algo dulce. Hubo esa frustración de empezar de nuevo. Me desanimé."	"I couldn't overcome my temptations … like sodas and deserts. I always needed something sweet. There was that frustration of starting all over again. I got discouraged." (Participant 11, Female).
Subtheme: Perceived Traditional Gender Roles	
"Pues no, no, no, que no es que sea machista, pero... pues no me veo haciendo aerobic. No, mejor me voy por las cosas que me gustan o algo así. A bicicleta o así, pero eso que el hula hula ofrece una rueda que tenían ahí de plástico. No es una. Pues no, yo no me veía en eso."	"It's not that I'm a chauvinist…but …I don't see myself doing aerobic exercises. No, I am better doing things I like or something like that… Riding a bicycle or something like that, but a hula hoop – this plastic ring they had there… that is not [something I like]. Well, no, I didn't see myself doing that." (Participant 3, Male)
"Algo que en lo que más podrían ayudar es continuar con la dieta. Mi esposa cocina para todos y estamos tratando de sacar algunas cosas. Hay momentos en que uno empieza a hacer algo y después de meses que terminamos olvidándonos de ello, o nos cansamos de la misma rutina. Volvemos a nuestras costumbres."	"Something that [my family] could help most with is to continue with diet changes. My wife cooks for everyone and we are trying to take out some [unhealthy foods]. There are times when one starts a [healthy habit] and after a few months we end up forgetting about it…or we get tired of the same routine. We go back to our old habits." (Participant 22, Male)
"Personalmente he tenido experiencias y con otras mamas que trabajan y cocinan que dicen que me rindo. Me salgo del trabajo, y trato de encontrar cosas para comprar que sean saludables y lo que a mi familia le gusta. Quiero decir, cuando es así, creo que es difícil hacer un cambio."	"I have personally had experiences, and with other mothers that both cook and work, that say they want to give up [making healthy changes]. I get out of work, and I try to find things to buy that are healthy and that my family likes. I mean, when it's like that, I think it's hard to make a change." (Participant 10, Female).
"Mi esposo se inscribió en el programa, pero con su trabajo no pudo ir a las clases. Pero yo le hacía comer sano, así que tenía que seguirnos el ritmo a los dos."	"My husband signed up for the program but with his job he couldn't attend the classes. But I would make him healthy meals, so I had to keep up [with diet changes] for the both of us." (Participant 1, Female)
Subtheme: Internalized Cultural Biases of Health Behaviors	
"Mi hija y yo vamos al gimnasio. Y es algo que había dejado de hacer en el pasado, lo había hecho antes y luego dejé de hacerlo y sobre todo, en nuestra	"My daughter and I go to the gym. And it's something that I had stopped doing in the past. I had done it before but then I stopped. In our culture it is not

© 2026 Tensun FA et al. *JAMA Network Open*.

cultura, no es algo muy normal asistir al gimnasio y hacer ejercicio.”	something normal to go to the gym and do exercise.” (Participant 8, Female).
“En nuestra cultura no es realmente normal ir al parque. Cansado, pero es algo tan simple, pero nuestra cultura no suele hacerlo. Ni siquiera el ejercicio. ¿Quién sabe cuánto tiempo hemos perdido en nuestra cultura en no hacer esos cambios?	“In our culture it’s not really normal to go to the park. It can be tiring but it’s something so easy, but our culture does not usually do it. Not even exercise. Who knows how much time we have lost in our culture by not making those [healthy] changes?” (Participant 1, Female).
“Ah, por ejemplo, hacer cambios es más difícil para toda la familia, y más aquí, que es un estilo de vida muy, muy rápido, muy acelerado. Tienes que seguir trabajando o algo así para tener cosas. Para vivir aquí. Es dificil.”	“Making changes is more difficult for the whole family, especially here, [in the United States] where the lifestyle is very fast paced, very accelerated. You have to keep working to have things [basic needs]. To live here. It’s hard.” (Participant 10, Female).
“Como hispanos, Latinos, algunos de nosotros no queremos ir al médico a menos que estemos enfermos, y realmente no hacemos ejercicio. No está en nuestra cultura.”	“As Hispanics, Latinos, some of us do not want to go to the doctor unless we are sick, and we really do not exercise. It’s not in our culture.” (Participant 17, Male).
**Theme 2: Family as Reciprocal Catalysts for Diabetes Prevention Action**	
Subtheme: PerceivedFamily Exercise Engagement	
“Por ejemplo, mis hijas, y mis nietos, me regalaban, por ejemplo, me regalaron un mat para hacer yoga con el nombre de cada uno de mis nietos, con sus manitas, me regalaron. Se llama pesas, que hicieron los niños, o sea, para motivarme a hacerle los ejercicios, porque no me gusta hacer ejercicio. ”	“For example, my daughter, my grandchildren, for example… they gifted me a mat to do yoga, with the names of my grandchildren and their little hands on it… to motivate me to do exercises because I don’t like exercising.” (*Participant 5, Female*).
“Yo haciendo los ejercicios. Mi niña, la chiquita que se ponía a un lado conmigo y ellas, (promotoras) nos dice, que todos pueden hacerlo. Estaba a mi lado y miraba. Me gustaron las clases; es realmente algo que nunca habría hecho… hacer ejercicio.”	“I would do the exercises. My girl, the little one, would be beside me and the [community health workers] would say that we can all do it. She would be by my side and would watch. I liked the classes; it is truly something I would have never done… to exercise. (Participant 7, Female).
“Lo que hacemos y ya hacemos es salir a caminar juntos. Oh, vamos a las montañas. Las montañas. Y vamos a caminar y andar en bicicletas.”	“What we [participant family] do and we now do is go outside and walk together. We go to the mountains, and we walk and ride bikes.” (Participant 3, Male)
“Caminar es algo que ahora hacemos como familia. Vamos al parque, también haciendo actividades que involucran comida. Todos estamos haciendo todo eso juntos ahora.”	“Walking is something we now do as a family. [We go] to the park, and we are also doing activities that involve food. All of us are doing all those things together now.” (Participant 22, Male)
Sí, podríamos salir a pasear todos juntos. Al fin de la semana, ve al parque o algo así. Pero ahora, es un poco complicado hacerlo juntos.”	“Yes, we could come to go out walk all together. At the end of the week, go to the park or something. But right now, it is a bit complicated to do it together. “ (Participant 4, Female).
Subtheme: Dietary Education Influences Family Eating Habits	
“Al principio, a mi familia no le gustaba la comida saludable que recibía. Pero siempre he agregado la nueva comida en sus comidas, pero esperaría. Luego, poco a poco, iría más en su comida.	“Initially my family didn’t like the healthy food I would get. But I have always added the new food [healthy food] in their meals, but I would wait. Then slowly I would add more in their meal.” (Participant 2, Female)
“Pues mis hijos sabían que estaba yo en estas clases y también, pues me recordaban. Acuérdate que eso no. Y así, y okay, y que íbamos a la tienda y ya estábamos agarrando, pues galletas o algo. Y decían no porque tiene mucha azúcar. Ellos nos ayudaban a escoger las cosas.”	“Well, my kids knew that I was participating in the classes, and they also would remind me – remember [you are not supposed to eat that]. When we would go to the store, and we would grab cookies for example, they would say “no because it has too much sugar”.

	They [Participant's children] would help us choose our food items." (Participant 1, Female)
"Bueno, teníamos esta costumbre de hacer tamales. Entonces, en lugar de comernos el tamal, así solo, era super delgadito como muy poquita. Pura masa y trabajarías para incrementar más verdura o reducir porciones. Por ejemplo, el tamal y ponía la verdura, una ensalada de lechuga con tomate. Ah sí. Ajá para que no fuera solo el puro tamal.	"Well, we [Participant's family] had this custom of making tamales. So, instead of eating just tamales, we would make the tamales much thinner... very tiny…pure dough, and we would work to add more vegetable or reduce portions. For example, having the tamal and adding a lettuce and tomato salad so it wasn't just the tamales." (Participant 12, Female).
"Mis hijos y yo compramos juntos y leemos las etiquetas. Diría también que, en ocasiones, mis hijos me han ayudado a hacer recetas."	"My kids and I shop together and read labels. I would say too, on occasion my kids have helped me make new [healthy] recipes." (Participant 11, Female).
"Traté de involucrarlo también, pero él no quiere comer saludable. Él dice que es demasiado difícil."	"I tried to involve him too. [Participant's spouse] but he does not want to eat healthy. He says it's too difficult to keep up with." (Participant 6, Female).
Subtheme: Collective Emotional Impact and Accountability	
"Me siento agradecida de que mi esposa me apoyara también. Ella no tiene ninguna enfermedad, pero me apoyó al estar conmigo al inscribirse y tomar clases juntos."	"I feel grateful that my wife supported me too. She doesn't have any illnesses, but she supported me by being with me when signing up and taking classes together." (Participant 18, Male).
"Mis hijos me preguntan; ¿llevaste a papá a clase también? Vamos a hacer cambios aquí. Queremos hacerlo hace un tiempo. No es fácil, pero lo estamos intentando. Me siento feliz. "	"My children ask me; did you take dad to class [National DPP class] too? We are going to make changes here! We have wanted to this for a while. It's not easy but we are trying together. I feel happy." (Participant 14, Female).
"Ellos son mi apoyo porque no me gusta salir sola... Se siente demasiado solo. Pero cuando están conmigo, puedo hacer mis ejercicios."	"[My family] are my support because I don't like going out by myself. It feels too lonely; but when they are with me, I can [go out and] do my exercises." (Participant 12, Female).
"Hay una complicación con nuestro horario de trabajo para mantener los hábitos saludables. Pero sí, se siente bien obtener ese ánimo para continuar."	"Currently, there is a complication with our work [Participant and Participant spouse] schedule to keep up healthy habits. But yes, it feels good to get encouragement to continue." (Participant 9, Female).
**Theme 3: Culturally and Linguistically Aligned Program Supports Participant**	
Subtheme: Perceived Support and Motivation from Community Health Workers	
"Sí, porque con cualquier pregunta uno tenía, nos lo preguntaríamos y bueno, estarían allí... eenseñándonos a todos y apoyándonos".	"Yes, because with whatever question we had, we [Participants] would ask ourselves and well, they [Community Health Workers] would be there... teaching us and supporting us." (Participant 6, Female).
Aprendí a hablar de mi frustración… pero en el programa, y todo lo que alguien necesita es alguien que lo escuché. Del mismo modo, ayuda a otras personas en el programa.	"I learned to talk through my frustration in the program. All anyone needs is someone to listen to them. In the same way, it helps other people in the program." (Participant 15, Female).

© 2026 Tensun FA et al. *JAMA Network Open*.

“Me siento más seguro con ellas, para hacerle preguntas más directamente a ellas. Cuando una persona te está mirando y escuchándote, hay más comprensión.”	“I feel more reassured by them, to ask questions more directly to them. It is easier to understand [what someone is teaching] when they are looking at you and listening to you.” (Participant 2, Female)
“Cuando terminamos el programa, había un seguimiento cada mes. Fue constante. Estaba motivada, pero cuando no había clases con ellos, las reuniones eran más lejanas. Empecé a rechazar de nuevo. Me desanimé y dejé de hacer ejercicio.”	“ When we [Participants] finished the program, there was follow up every month. It was pretty consistent. From there, I was pretty motivated, but when there were no classes with them [Community Health Workers], the meetings were more far out. I started to decline again. I got discouraged and did not exercise anymore.” (Participant 13, Female).”
Subtheme: Culturally and Linguistically Concordant Information is Essential	
“Sí, trabajaron muy duro con nosotros para enseñarnos cómo comprar alimentos en el mercado y leer etiquetas como alimentos que tienen demasiado azúcar.”	“Yes, [Community Health Workers] worked very hard with us to teach us how to buy food in the market and read labels like foods that have too much sugar.” (Participant 19, Male)
“Nos enseñaron a preparar la comida, pero de una manera más saludable… Así que sí, me ayudó, porque me dio opciones para pensar y entender, puedo comer esto, pero puedo hacerlo saludable”	“They [Community Health Workers] taught us how to prepare food, but in a healthier way…So yes, it helped me, because it gave me options to think and understand - I can eat this, but I can make it healthy.” (Participant 11, Female)
“Nos dieron buenos consejos para cuando vamos a la tienda, como leer etiquetas y contar calorías.”	“They [Community Health Worker] gave us good tips for when we go to the store, like reading labels and counting calories.” (Participant 12, Female).
“Ellas dieron información. De todas partes. Lo más importante era cómo se causaba la diabetes, ¿Quién la contrae? Las diferencias entre diabetes y prediabetes. Bueno, nos lo explicaron de una manera muy fácil de entender.”	“They gave information…the most important was how diabetes was caused, who gets it? The differences between diabetes and prediabetes. Well, they explained to us in a way that was very easy to understand.” (Participant 9, Female).
“Debe haber más información que se le diga al la familia para claridad. Leer las etiquetas fue difícil. Para explicar.”	“There should be more information said to the rest of the family for more clarity. Label reading was hard for me explain.” (Participant 7, Female).
Subtheme: Perceived Peer Support through Shared Experiences	
“También fue muy útil tener a los otros participantes allí. Ellos también compartieron sus experiencias. Realmente nos sentimos como una comunidad que se apoya unos a otros.”	“It was also very helpful to have the other participants there. They also shared their experiences. We truly feel like a community that supports one another.” (Participant 18, Male).
“Había gente como yo en el programa…tratando de hacer las mismas cosas. Bueno, todos teníamos las mismas condiciones, y podíamos hacerlo... apoyándose unos a otros para continuar.”	“There were people like me in the program... trying to do the same things. Well, we all had the same conditions, and we could do it... supporting one another to continue.” (Participant 8, Female)
“Escuché a otras personas que tienen problemas similares y me di cuenta de que no era solo yo. Yo puedo hacerlo.”	“I listened to other people that have similar problems as me and I realized that it’s not just me [I am not alone]. I can do it.” (Participant 20, Female)
“Comenzamos a hacer la nutrición, pero yo no perdía peso y algunos compañeros también. Y algunos de nosotros éramos iguales. Así que eso fue difícil.”	“We started doing the nutrition but I was not losing weight and some other peers were losing weight. And some of us were the same. So that was difficult.” (Participant 16, Female).”
**Theme 4: Reflection on Personal Motivation for Behavior Change**	
Subtheme: Family Diabetes Lived Experience Awareness and Motivation	

© 2026 Tensun FA et al. *JAMA Network Open*.

"Mi mamá tenía diabetes. Tengo mucha familia con diabetes y la mayoría de mis hermanas son diabéticas porque no se cuidan solas. Tengo que estar saludable."	"My mom had diabetes. I have a lot of family with diabetes and majority of my sisters are diabetic because they don't take care of themselves. I have to be healthy." (Participant 14, Female).
"La mamá de mi esposo tiene diabetes, ¿verdad? Entonces deberíamos tratar de mejorar eso porque tal vez somos propensos. Más tarde, si no nos cuidamos, también estamos desarrollando diabetes. Así que sí, como juntos, ¿verdad? Para intentarlo"	"My husband's mother has diabetes, right? So, we should try to improve [our health] because we are prone to diabetes. If we don't take care of ourselves, later we will also develop diabetes. So, we should try together." (Participant 20, Female).
"Yo participé porque mi familia, todos tienen diabetes. La mayoría de mis hermanos tienen antecedentes familiares de diabetes. ¿Y yo? En otras palabras, me preocupo por mi salud. Lo primero es la salud."	"I participated because my family all have diabetes. The majority of my siblings carry a family history of diabetes…In other words, I do care about my health. The most important thing is health." (Participant 17, Male)
"Vi a mi mamá sufrir diabetes y finalmente le quitó la vida. Así que sí, eso me motiva."	"I saw my mom suffer from diabetes and eventually it took her life. So yes, that motivates me." (Participant 6, Female).
Subtheme: Desire to Model Lifestyle Behaviors	
"Yo soy quien cocina. Ellos comen lo que yo trato de comer... como las verduras."	"I am the one who cooks. They [Participant family] eat what I try to eat... like vegetables." (Participant 4, Female).
"Quiero ser un buen ejemplo para ellos, incluso si soy la abuela, puedo estar saludable. Puedo hacer cambios. No importa la edad que tenga para ser activo… un buen ejemplo para ellos."	"I want to be a good example for them even if I'm the grandmother, I can be healthy. I can make changes. It does not matter what age I am to be active... a good example for them." (Participant 5, Female).
"Quiero seguir haciendo ejercicio y tener un estilo de vida saludable para apoyar a mis hijos. Un ejemplo es que, si quiero cambiar eso en mis hijos, necesito empezar primero, y es de esa manera que nos apoyamos mutuamente…y ayudándonos unos a otros."	"I want to continue doing exercise and having a healthy lifestyle to support my kids. An example is that, if I want to change that in my kids, I need to start first, and in way we support each other, helping one another..." (Participant 8, Female).
"No quería hacerlo por mí, sino por mi familia. Con el tiempo, nos ayudamos a resistirnos a comer comida chatarra."	"I didn't want to do it for me but for my family. Eventually we helped each other resist eating junk food." (Participant 12, Female).
"Estamos trabajando juntos. Pero con él, se rindió. Ya no quería comer sano. Lo intentó de nuevo, pero luego comió más tortillas. ¿Yo? No. Yo preparo mi propia comida. Empezó de nuevo con eso."	"We are working together.[Participant and Participant's Spouse] But with him [Participant's spouse], he gave up. He did not want to eat healthy anymore. He tried to again but then he ate more tortillas. Me? No. I make my own food. He started over with that. [Healthy eating] (Participant 1, Female)."
Subtheme: Perceived Mental Health and Spiritual Appreciation	
"De esto, aprendo a decirme a mí mismo y a la vez hacerles conscientes de que si continúan con una mala dieta puede ser una situación de salud. Pero todos nos sentimos bien mentalmente haciendo cosas juntos."	"[From the National DPP] I learn to tell myself and at the same time make them [Participant's family] aware that if they continue with a poor diet, they will have health risks. But we all feel mentally good doing things together."(Participant 21, Male).
"La verdad es que a veces, nosotros como humanos no queremos entender y no nos damos cuenta - Podemos prevenir enfermedades si realmente ponemos nuestra mente. "	"The truth is that sometimes, we as humans do not want to understand [health] and we don't realize - We can prevent diseases if we really put our minds into it." (Participant 15, Female).
"Algo positivo que me sigue motivando es el yoga y tener a mi familia ahí. La mejor parte fue que siempre estuvieron ahí para mantenerme en forma y me ayudó mucho mentalmente."	"Something positive that continues to motivate me is yoga and having family there for me. The best part was that they [Participant family] were always there to keep me fit and it helped me a lot mentally." (Participant 10, Female).

© 2026 Tensun FA et al. *JAMA Network Open*.

“A veces podemos caer mentalmente, pero uno necesita pedirle mucho a Dios que nos ayude a superarlo. Afortunadamente superé eso.”	“Sometimes we might fall mentally but one needs to ask God to help us get through it. Thankfully I overcame [those diabetes related challenges].” (Participant 15, Female)
“Cuando uno se queda atrás en eso. ¿Cómo se siente uno? Bien deprimido o algo así. Y sientes que no puedes hacerlo. Quieres volver a tu vida. Antigua. con mala comida, dieta y cosas asi. "	“When one falls behind on that [Continuing healthy habits]. How does one feel? Well depressed or something. And you feel like you can’t do it. You want to go back to your old life with bad food, diet, and things like that.” (Participant 16, Female).”
**Theme 5: Desired Components of Family Inclusion**	
Subtheme: Perceived Role in Knowledge for Family Participation	
“Me hubiera gustado involucrarme más en la comprensión de los problemas... Como la comprensión, como para mí, estaba tomando clases y también se las explicaba a mi esposo, pero él habría entendido más escuchando en la clase.”	“I would have wanted to get more involved with understanding the problems [diabetes]…. I was taking classes, and I would explain them to my husband too. But he would have understood more listening in the class.” (Participant 6, Female).
“Bueno, más que nada, quiero que estén informados. Porque me dijeron que ya estaba informado. No es lo mismo que yo venga y les diga la información”	“Well, more than anything, I want them [Participant family] to be informed. Because they told me that I was already informed. It is not the same for me to come and tell them the information.” (Participant 22, Male).
“Todos tenemos que entender por qué hay que comer bien. Y que comer algunos alimentos nos perjudican también.”	“We need to all understand why we should eat healthy food. As well as learning that some foods may harm us too.” (Participant 19, Male)
“La única manera en que su familia puede ayudarlo es tomando conciencia, es decir, informándose a sí mismos para que también tomen conciencia de la diabetes.”	“The only way your family can help you is by becoming aware, that is, informing themselves so they become aware of diabetes too.” (Participant 5, Female).
“Una de esas sesiones, deberían haber invitado a la familia. Hubiera sido genial si hubieran sido invitados a obtener esa información.”	“One of those sessions, they should have invited the family. It would have been great if they were invited to get that information.” (Participant 12, Female).
Subtheme: Structured Family-Friendly Wellness Activities	
“Queremos hacer mucho ejercicio. Mis hijas tratan de mantenerse activas al gimnasio, yo trato de hacer batidos. Ellas van más que yo, porque para mí es tener tiempo. Y dónde vivir. Voy a clases de zumba. Pero estaría bonito hacerlo juntas.”	“We want to do a lot of exercise. My girls try to stay active going to the gym. They go more than me, because the problem for me is having time, and where I live. I go to Zumba classes. But it would be nice to do it together.” (Participant 14, Female).
“Queremos implementar más en el verano, cuando hace más calor. Para ir más al parque, andar más en la bicicleta. Quiero seguir con el ejercicio para continuar con las metas y estar activa.”	“We [Participant family] want to do more in the summer when it gets warmer… to go to the park more, bike more. I want to continue with exercising to continue with my goals and stay active.” (Participant 4, Female).
“Me gustaría que mi esposo estuviera más involucrado y me acompañara a hacer ejercicio. Pero ahora, como digo, no puedo obligar a una persona a hacer lo que quiero, ¿Verdad?”	“I would like my husband to be more involved and accompany me to exercise [but…] I think, I can’t force a person to do what I want, right?” (Participant 8, Female).
"Sí, podríamos venir a salir a caminar todos juntos al final de la semana ir al parque o algo así. Pero ahora es un poco complicado hacerlo juntos.”	“Yes, we [Participant’s family] could come to go out walk all together at the end of the week go to park or something. But now it is a bit complicated to do it together. “(Participant 6, Female).
Subtheme: Preventing Diabetes Among Children	
“Un día con suerte, puedo inscribir a mis hijos en el programa correctamente. Al igual que incluirlos es clave, para que podamos tener una charla de que estamos todos juntos, ¿verdad?”	“I hope one day I can sign up my children for the program. Including them is key, so we can say we are all in it together, right?” (Participant 11, Female).

© 2026 Tensun FA et al. *JAMA Network Open*.

“Me gustaría que mis hijos estuvieran motivados y aprendieran un poco de sus snacks.”	I would like my kids to be motivated and learn a bit about their snacks. (Participant 10, Female).
“Tenían el programa para niños, pero ya no lo hacen. Pero me gustaría que fuera más de eso. Como las clases de nutrición, hacer clases de comida, porque aprendí mucho allí.”	“The [Diabetes Program] had the program for young children, but they don’t have it anymore. But I would like it to be more of that like nutrition… classes, because I learned a lot there.” (Participant 7, Female).
“Nuestros hijos siempre están aprendiendo, y siempre trato de enseñarles cosas. Espero que en el futuro también puedan enseñar este programa a los niños, y enseñarles cosas como comer sano, hacer ejercicio y caminar. Todo eso es importante.”	“Our children are always learning, and I always try to teach them things. I hope in the future they can also teach this program to kids, and teach them things like eating healthy, doing exercise, and walking. All of that is important.” (Participant 1, Female).
“Bueno, cuando era una niña… comes cuando estás lleno y tener ese hábito es difícil de quitar. Te llenas hasta que estás satisfecho. Enseñar eso a los niños puede ser difícil".	“ Well when I was a little girl…You eat when you’re filled up and having that habit is hard to take away. You get full until you are satisfied. Teaching that to kids may be hard.” (Participant 13, Female).

© 2026 Tensun FA et al. *JAMA Network Open*.
