# Supplementary material for: Understanding Latino Individual and Family Perspectives in a National Diabetes Prevention Program
Source: JAMA Netw Open. 2026 Apr 2;9(4):e264780. doi: 10.1001/jamanetworkopen.2026.4780 (PMC13047464; doi:10.1001/jamanetworkopen.2026.4780)
Supplement: Supplement 2. — Data Sharing Statement [file jamanetwopen-e264780-s002.pdf]

Data Sharing Statement

Tensun. Understanding Latino Individual and Family Perspectives in a National Diabetes Prevention Program. *JAMA Netw Open*. Published April 02, 2026. doi:10.1001/jamanetworkopen.2026.4780

Data
Data available: No
